# Identifying the challenges and opportunities of PCOS awareness month by analysing its global digital impact

**DOI:** 10.3389/fendo.2023.1109141

**Published:** 2023-03-02

**Authors:** Kashish Malhotra, Carina Synn Cuen Pan, Meri Davitadze, Team PCOS SEva, Justin J. Chu, Punith Kempegowda

**Affiliations:** ^1^ Department of Medicine, Dayanand Medical College and Hospital, Punjab, India; ^2^ College of Medical and Dental Sciences, University of Birmingham, Birmingham, United Kingdom; ^3^ Department of Endocrinology, Georgian-American Family Medicine Clinic ‘Medical House’, Tbilisi, Georgia; ^4^ Department of Endocrinology and Diabetes, University Hospitals Birmingham National Health Services (NHS) Foundation Trust, Birmingham, United Kingdom; ^5^ Institute of Metabolism and Systems Research, University of Birmingham, Birmingham, United Kingdom

**Keywords:** PCOS, polycystic ovary syndrome, PCOS awareness month, social media, mental health, collaboration, global equity

## Abstract

**Background and objective:**

Although significant resources are invested each September for PCOS Awareness Month campaign, there are no studies measuring its impact. We evaluated the digital impact of PCOS Awareness Month, common themes and associated topics, top influencers, and global equity of influence during the PCOS Awareness month.

**Methods:**

In this serial cross-sectional analysis, we studied the outputs from Symplur^®^ to study the total impressions of #PCOS on Twitter^®^. We tracked the hashtags—#PCOS, #PCOSawarenessmonth, #PCOSawareness—and a search query— “#PCOS OR #PCOSawarenessmonth OR #PCOSawareness”—using Sproutsocial^®^ to study the total number of tweets related to PCOS Awareness Month. Network analysis was done using SocioViz^®^ to identify common themes and associated topics. Using SymplurRank^®^ machine learning algorithm, the top 10 #PCOS influencers were identified based on the number of mentions received. Google^®^ Trends was used to study the web and news search popularity over the last 10 years beyond social media platforms.

**Results:**

An overall upward trend in the digital impact of PCOS awareness was noted since 2017. While the top themes associated with PCOS (insulin resistance, depression, anxiety, menopause, hormones, infertility) remained the same in 2021 and 2022, newer themes emerged in the latter year suggesting the need for ongoing review. News outlets were the most influential organisations during PCOS Awareness Month in both years of study. Seven of the top 10 users were the same in both years. Limited engagement from African, Asian, South American, and non-English speaking European countries was seen on Google Trends analysis.

**Conclusion:**

Active involvement from various stakeholders of PCOS Awareness Month has shaped it into an effective strategy to raise awareness with social media playing a crucial role in amplifying the message. Our findings also provide an opportunity to understand the current perceptions and expectations amongst the public, which can influence future healthcare investment and research.

## 1 Introduction

PCOS is a common endocrine disorder with a global prevalence of 8-13% in women of reproductive age group (1). PCOS Challenge, a patient support organisation based in the USA, successfully led an advocacy effort for PCOS to be recognised by US legislation in 2017 (2, 3). Thereafter, September was federally designated as the month to raise awareness for PCOS in the USA, henceforth marking September 2017 as the first-ever PCOS Awareness Month (4). The vision of PCOS Awareness Month includes improving the quality of life of people with PCOS, promoting research into developing better diagnostic and treatment care plans, and advocating for international support for PCOS (5, 6).

Throughout PCOS Awareness Month, various organisations, and self-help groups campaign for PCOS by conducting symposia and conferences with involvement from media outlets, notable individuals, and the public. These events bring together healthcare professionals and members of the public to learn more about PCOS. This enables the formation of supportive communities, which leads to empowerment and translation to improved care for patients with PCOS (7, 8).

There are limited studies on the impact of such healthcare awareness initiatives (9, 10). While Malhotra et al. have previously published their work on the digital impact of World Hypertension Day (11), we couldn’t find any similar studies about PCOS. There is a potential to use such events to identify the common themes that are discussed to identify gaps and opportunities to increase the effectiveness of PCOS Awareness Month. Also, it is important to identify key influencers as they have the power to control the narrative and outcome of such events. Finally, identifying the geographical equity of the awareness month is important to assess if the information shared is not skewed by perspective of one group or region. Therefore, we designed this study with the following objectives.

1. To study the digital impact of PCOS Awareness Month initiative
2. To identify the common themes and associated topics during the awareness month
3. To identify the top influencers during the awareness month
4. To study the global equity of influence during the awareness month

## 2 Methods

This study was done by the PCOS SEva team from August 2021 to October 2022 which is a collaboration of early career and senior researchers who have a common objective to improve the services provided for people with PCOS. The authors of this article liaised extensively with all members of the PCOS SEva (Polycystic Ovary Syndrome Service Evaluation) team to refine the methods to ensure authenticity and reliability.

### 2.1 Digital impact of PCOS Awareness Month

We studied the total tweets and impressions for #PCOS on Twitter using Symplur^®^ (Symplur LLC, Los Angeles, CA, USA) (12). “Impressions” were defined as the product of all tweets by each participant and the number of followers that each participant currently has, then summed up for the total number of participants. To study the activities related to PCOS Awareness Month, we tracked hashtags associated with the awareness month. Hashtags are commonly used in social media platforms to advertise events due to their universal reach and accessibility. When a hashtag is added, it is linked to all other posts that include it, thereby giving one’s post context and allowing the public to follow topics they are interested in. Studies assessing the impact of healthcare initiatives, such as Breast Cancer Awareness Month (13) and global surgery (14) through social media hashtagging, have shown promising results in identifying barriers and strengthening collaboration. We adopted a similar strategy and evaluated the global digital impact of PCOS Awareness Month on Twitter^®^ by tracking hashtags—#PCOS, #PCOSawarenessmonth, #PCOSawareness—and a search query—”#PCOS OR #PCOSawarenessmonth OR #PCOSawareness”—using SproutSocial^®^ (Sprout Social Inc., Chicago, Illinois, USA) (15). This search query will henceforth be referred to as “PCOS Hashtags” in this article. A search query using the Boolean operator “OR” helps to connect two or more similar concepts and broaden the results by including any of the mentioned search terms in the result (16). The total number of tweets and tweets per day with PCOS hashtags were extracted from 1^st^ August to 31^st^ October 2014-2022. Year-on-year trends were measured which indicated an increase or decrease in hashtag usage during the reporting period. The data of the above-mentioned hashtags (#PCOS, #PCOSawarenessmonth, #PCOSawareness) were also separately extracted for those users with a verified badge on Twitter who have notably more following and influence (17). These data extractions were done prior to the Twitter acquisition.

### 2.2 Identifying the common themes and associated topics during the awareness month

Social network analysis of “PCOS Hashtags” was done using SocioViz^®^ (Via Bionde, Verona VR, Italy) on the last day of the PCOS Awareness Month for the most popular and recent tweets to deduce common themes. Network analysis provides the capacity to estimate complex patterns of relationships and gives insights into useful information about engagement, reach, and interactions in an environment (18, 19). The search setting was optimised to include both popular and real-time results. ForceAtlas2 was used as the layout for network analysis (20). The top 100 related popular hashtags were extracted, and hashtag network analysis was performed where each hashtag was represented with a circle (a node) and was connected to another hashtag when there was a co-presence in the same tweet (21). Different colours represented different clusters of arguments that frequently go together. We compared these results to the 2021 data which we collected similarly.

### 2.3 Identifying the top influencers during the awareness month

Using SymplurRank^®^ machine learning algorithm (12), the top 10 #PCOS influencers during PCOS Awareness Month were identified based on the quality of the number of mentions received. The quality of a mention was determined by the account’s influence, its healthcare stakeholder status, and its overall influence in healthcare social media. This was done to minimise the manipulation of simplistic metrics, such as the number of mentions, tweets, followers, etc.

### 2.4 To study the global equity of influence during the awareness month

Google^®^ Trends was used to study and track the web and news search popularity of queries “PCOS Awareness Month”, “PCOS Awareness” and “PCOS” for the last 10 years globally (from August 2012 to October 2022) to get an overall idea of the internet search trends beyond social media platforms. In all the trackers and analyses, no language or geographical preference was set.

## 3 Results

### 3.1 Digital impact of PCOS awareness month

**Figure 1:**
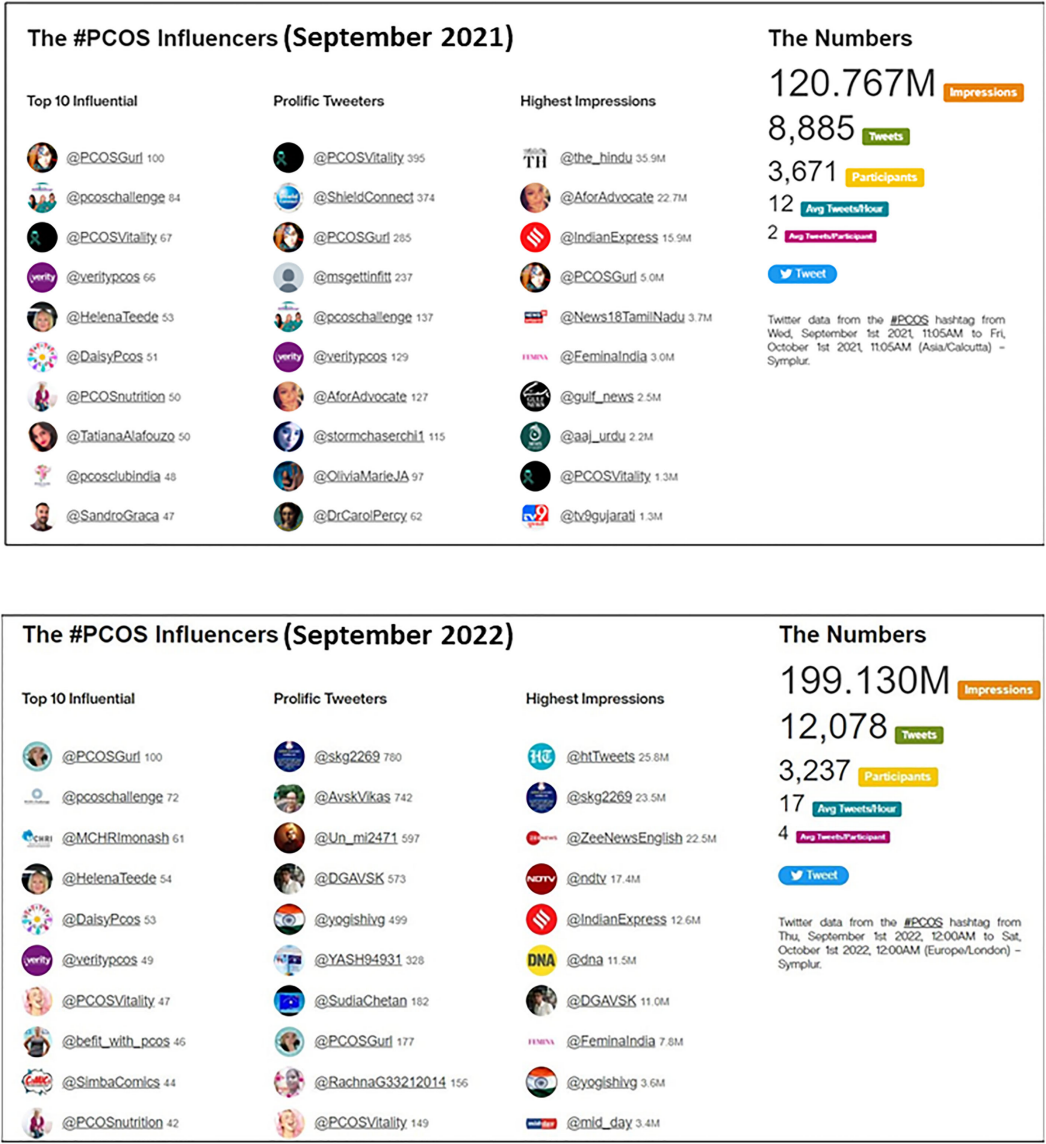
Top Twitter influencers and the global digital impact of #PCOS.

**Figure 2:**
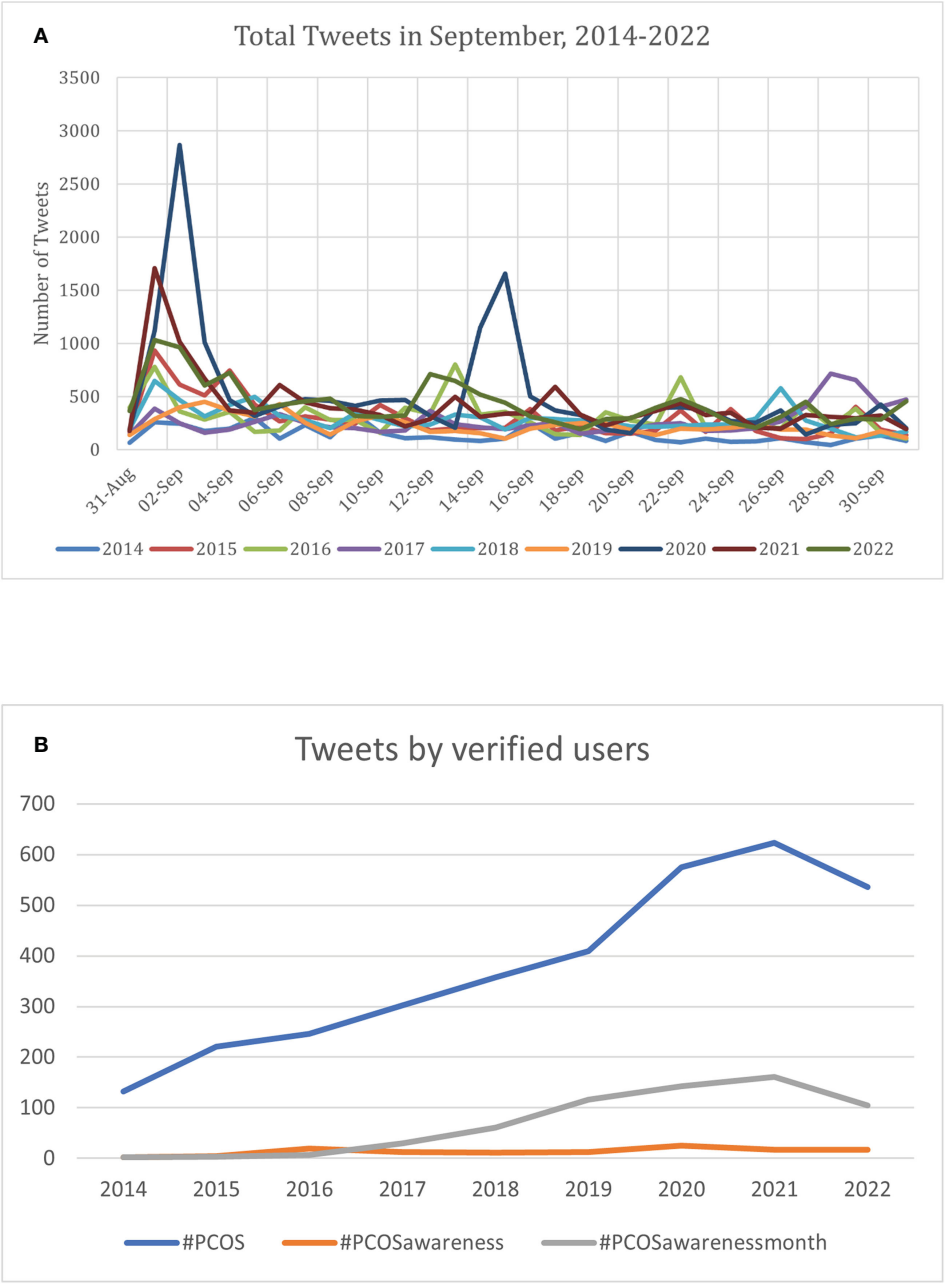
**(A)** Daily trend of the tweets posted each year with the search query “PCOS Hashtags” from 2014-2022. **(B)**. Yearly trends of the total tweets posted by verified users during PCOS awareness month using the hashtags- #PCOS, #PCOSawareness, and #PCOSawarenessmonth from 2014-2022.

PCOS amassed 199.13 million impressions in September 2022 which shows an increasing trend over the total impressions received in September 2021 (120.828 impressions) (**Figure 1**). The highest spike of total tweets (16,465) for “PCOS Hashtags” in September was noted in 2020 which also highlights the highest yearly increase (136.1%) in total tweets since September 2014 (**Table 1**). An initial spike in total tweets was seen at the start of September followed by a spike in mid-September with limited involvement for the rest of the month in both 2021 and 2022 (**Figure 2A**). Though the overall number of tweets from verified users using #PCOS and #PCOSawarenessmonth have been increasing (**Figure 2B**), the total number of tweets by verified users is a small proportion of the total tweets posted. For instance, in September 2022, tweets by verified users accounted for 4.5% of the #PCOS tweets, 0.9% of the #PCOSawareness, and 2.9% of the total #PCOSawarenessmonth tweets.

**Table 1:** Total number of tweets posted on yearly basis in the PCOS Awareness Month (September) with the search query “PCOS hashtags”.

Year	Total Tweets using “PCOS Hashtags” posted in September	Yearly Percentage change
2014	4,288	–
2015	9,182	114.1%
2016	9,599	4.5%
2017	8,115	-15.5%
2018	8,910	9.8%
2019	6,975	-21.7%
2020	16,465	136.1%
2021	12,838	-22.0%
2022	12,973	1.1%

In Google Trends web-search analysis, spikes were noted for the search query “PCOS Awareness Month” in September followed by a sharp decline in October each year. The highest interest was recorded in September 2020. The overall volume of news published for the search query “PCOS” in the month of September showed an increasing trend.

### 3.2 Common themes and associated topics

**Figure 3:**
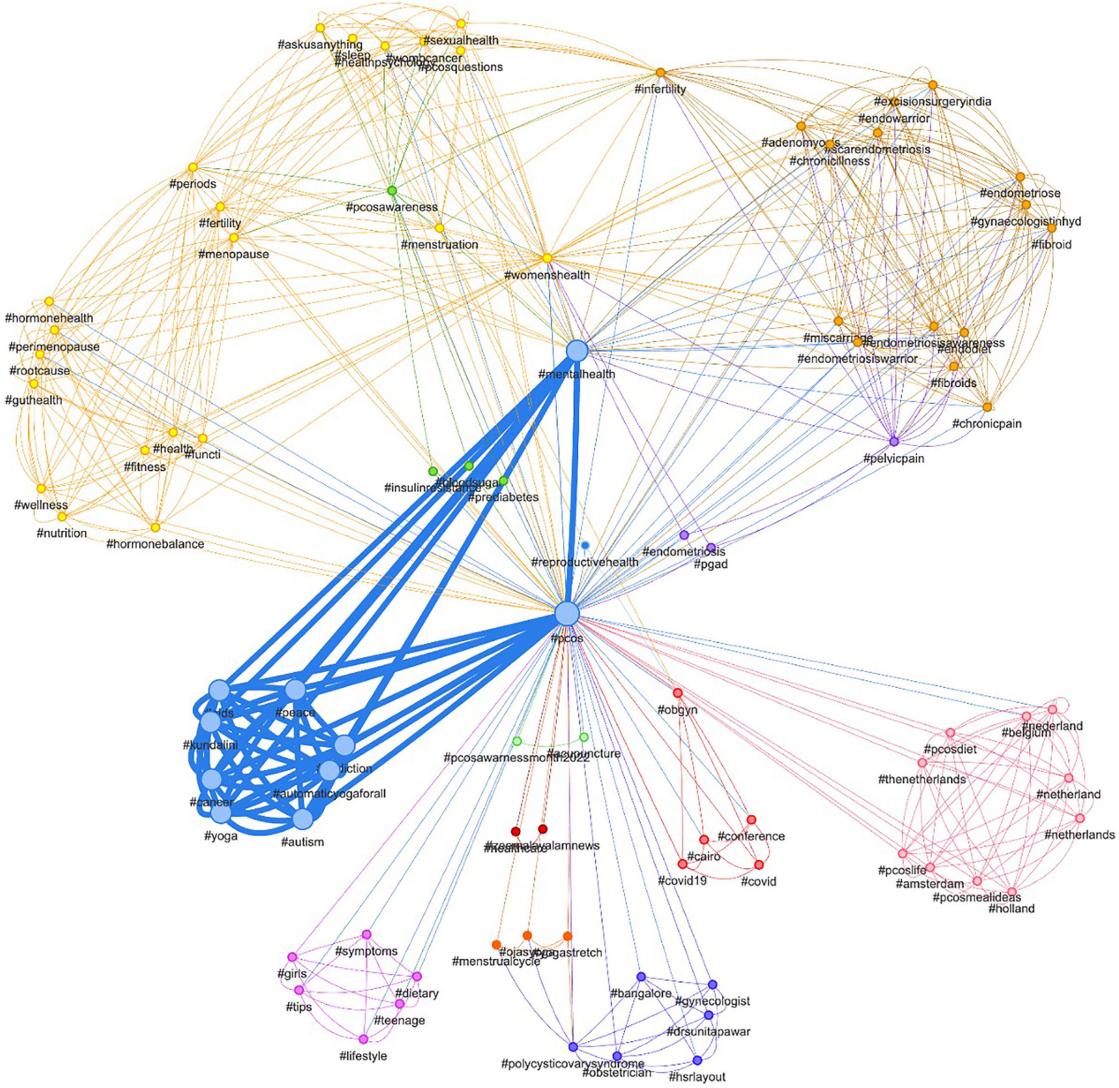
Network analysis of the relation between various hashtags and “PCOS Hashtags” in September 2022.

In September 2021, the top 10 common themes associated with PCOS awareness month were women’s health, mental health, infertility, nutrition, cardiovascular diseases, digital health, pregnancy, diabetes, self-care, and menstrual health (**Supplementary 1**). In September 2022, the common themes were mental health, insulin resistance, hormone balance, menopause, sexual health, infertility, chronic pain, endometriosis, yoga, and autism (**Figure 3**).

### 3.3 Top influencers during PCOS awareness month in 2022

Seven of the top 10 users amassing the highest impressions in both September 2021 and September 2022 were of news media outlets (**Figure 1**). Amongst the top 10 most influential accounts in September 2021, 5 were of PCOS researchers and/or advocates (@PCOSGurl, @HelenaTeede, @PCOSnutrition, @TatianaAlafouzo, @SandroGraca) followed by accounts of reputable organizations (@pcoschallenge, @PCOSVitality, @veritypcos, @DaisyPcos, and @pcosclubindia).

Amongst the top 10 most influential accounts in September 2022, 4 were of PCOS researchers and/or advocates (@PCOSGurl, @HelenaTeede, @befit_With_pcos, @PCOSnutrition) followed by accounts of reputable organizations (@pcoschallenge, @MCHRImonash, @DaisyPcos, @veritypcos, @PCOSVitality, and @SimbaComics). Amongst the most influential accounts, seven of the top 10 users were the same as last year.

### 3.4 Geographical equity

**Figure 4:**
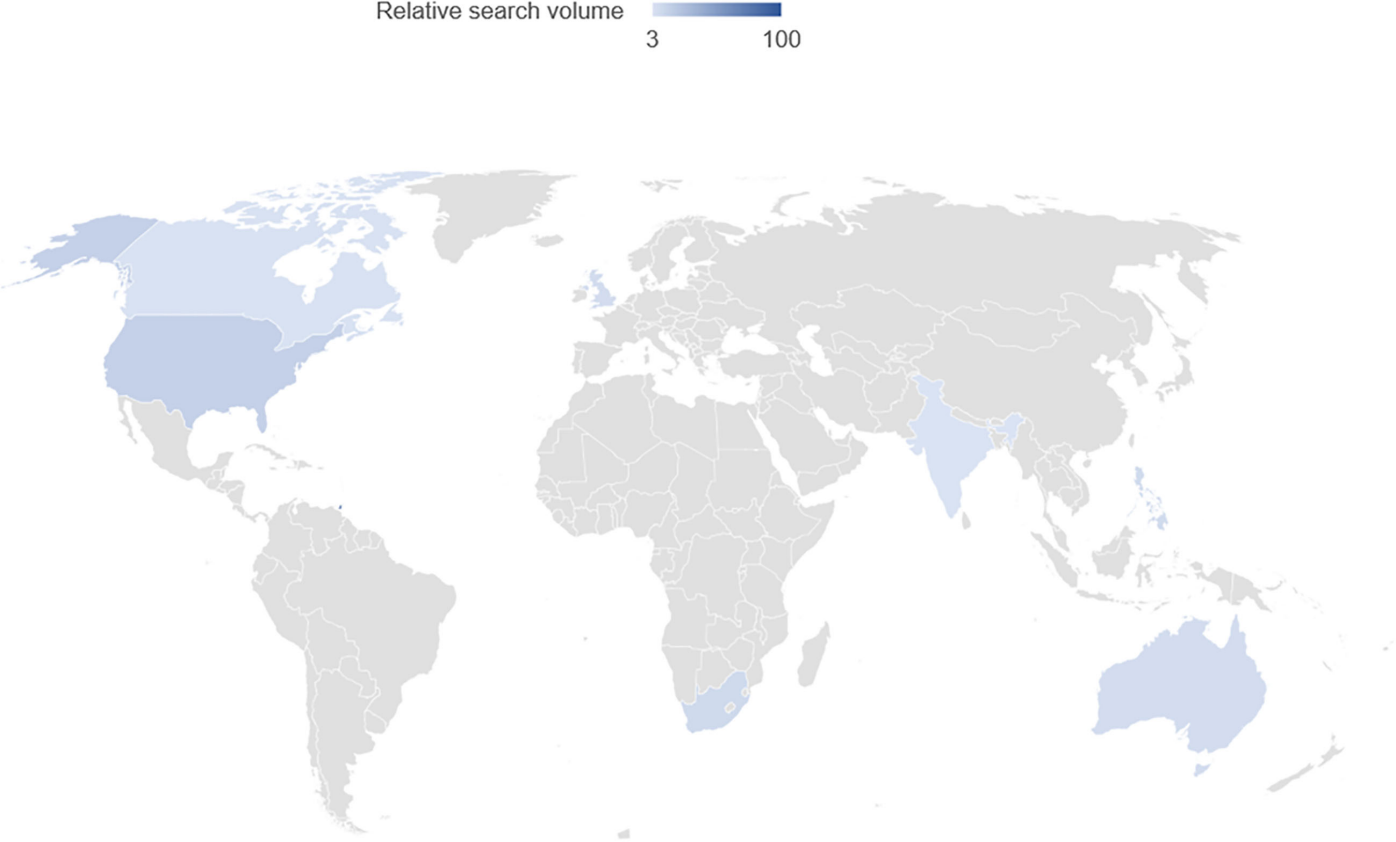
Demographics of web search interest by region for the search terms “PCOS Awareness month” from August 2012 to October 2022. This world map is for diagrammatic representation only and doesn’t purport to be a political map of any country.

On Google trends analysis, we noted involvement from several high- and middle-income countries including USA, UK, Australia, India, Canada, South Africa, Trinidad & Tobago, and Philippines. However, there was limited engagement in African, Asian, South American, and non-English speaking European countries (**Figure 4**).

## 4 Discussion

To the best of our knowledge, this is a first-of-its-kind assessment of the impact of awareness programmes in endocrine-related conditions. We explored the impact of PCOS Awareness Month by observing monthly trends as well as yearly trends. We utilised several online tools i.e. Symplur^®^, SocioViz^®^, SproutSocial^®^, and Google^®^ Trends with algorithms that enabled us to screen for all types of languages to reduce selection bias.

An overall upward trend in PCOS awareness suggests the effectiveness of active involvement from various stakeholders and legal recognition of the cause. However, legal recognition may not always lead to increased impact as seen in the case of Hernia Awareness Month (22). Furthermore, increased engagement may not ultimately lead to increased positive impact due to various limitations such as misinformation spread and stigmatising online coverage. The spike in activity in 2020 possibly coincides with the increased global online activity with the pandemic when there was limited face-to-face and other forms of interaction. Of note, as the total number of tweets posted in 2021 and 2022 were less than in 2020, it reflects on the continued support and engagement needed from the community to have surging reach.

Investigating other hashtags related to PCOS enabled us to determine the most associated topics through keyword network analysis. While the most common hashtags associated with PCOS Awareness Month remained the same in 2021 and 2022, there were a few other topics unique to each year. The discussion around yoga and autism are important to recognise so these can be addressed accurately minimising any misinformation. A few other hashtags such as infertility and mental health supports the need for holistic approach in the management of PCOS. This finding is in line with our previous work where people with PCOS highlighted the importance of holistic approach to the condition (23). These associations can also help guide future healthcare investments and research by identifying the gaps in perception of PCOS by healthcare professionals and the people affected by PCOS.

In our previous work, we highlighted the importance of influencers and their perspectives about PCOS (24). News media outlets play a crucial role in dissemination of information online and several media outlets participated in posting about PCOS Awareness Month and reaching a wide audience. Consistently increasing involvement of verified users shows an increased interest in the PCOS awareness month amongst the notable personalities and organisations. However, due to the recent policy changes to verified users after the Twitter acquisition may require methodological changes to identify notable influencers.

Our findings show tweeting about PCOS awareness was largely a singular event, at the start and mid of the month, and not consistent throughout the month, similar to a study analysing the social media trends of Breast Cancer Awareness Month (13). We note the top influencers led support for the PCOS Awareness Month year-on-year sustaining the conversations related to PCOS. This finding is contrasting to the evaluation of other similar initiatives where the awareness months had limited reach with declining trends (22, 25). Therefore, there is an opportunity for potential collaborations between these key players and organisations so a collective effort can amplify the efforts to reach a larger and more diverse audience.

More active involvement and partnerships are needed with other countries especially African, Asian, South American, and non-English speaking European countries to increase the reach of PCOS awareness campaign globally. Endorsement from the current key players to target and encourage participation from the African, Asian and other underrepresented audiences using native language or local culture-specific keywords or hashtags may help in increasing engagement. The limited impact of this event in Africa is consistent with the current literature evaluating the impact of other awareness events (11, 26). While we were able to identify several news articles and social media posts in native languages, the lack of overall engagement highlights the need for targeted action-driven campaigning to foster supportive communities and increase involvement from underrepresented communities. Promoting regional integration with sustainable economic development, social development, and strengthening local institutions with equitable partnerships may help to increase diversity and inclusion (27).

### 4.1 Limitations

The online tools used in the study have proprietary algorithms. However, we used a combination of multiple tools to yield cumulative results to overcome bias from an individual social media assessment tool. Our study didn’t include data from private profiles where data were not accessible. We analysed data from Twitter in this study and hence organisations or users that do not use Twitter as their main social media platform to advertise for events related to PCOS awareness were not accounted for. As other social platforms like Facebook, Instagram, and LinkedIn do not provide general access to their application programming interface for research, we were unable to include data from these platforms. We compensated this by studying trends on Google, one of the largest internet search engines. Other hindrances in data sampling include the specificity of hashtag screening. Intentional or unintentional variance from the hashtag such as from a spelling error may result in the data not being captured. Despite these limitations, our data show that PCOS Awareness Month as an initiative has a significant impact on a global scale.

## 5 Conclusions

There is a consistent increase in digital impact of PCOS Awareness Month, suggesting the initiative to be an effective strategy to raise awareness about PCOS. The common themes generated by public during PCOS Awareness Month may help identify potential areas of knowledge gap and future research interest. Similar top influencers over the years suggest an opportunity to collaborate to amplify the message. There is a need to enhance digital engagement with PCOS Awareness Month in African, Asian, and South American continents to establish global equity in the message shared online.

## PCOS SEva team collaborators

Justin J. Chu, Helena K. Gleeson, Meghnaa Hebbar, Sindoora Jayaprakash, Halimah Khalil, Tejal Lathia, Eka Melson, Alisha Narendran, Lynne Robinson, Chitra Selvan, Jameela Sheikh, Saskia Wicks, Nawal Zia, Michael O’Reilly and Wiebke Arlt.

## Data availability statement

The original contributions presented in the study are included in the article/**Supplementary Material**. Further inquiries can be directed to the corresponding author.

## Author contributions

KM and PK conceptualised the study. KM and CP were involved in all stages of the study since conception contributing equally to this work and share first authorship. MD conducted the searches alongside KM and CP and screened the data to ensure appropriateness and authenticity. PK supervised all stages of the study and is the senior author of this article. Members of the PCOS SEva team provided substantial contributions to the conception and design of the work study and were involved in discussions at all stages of the study. The PCOS SEva team includes Justin J. Chu, Helena K. Gleeson, Meghnaa Hebbar, Sindoora Jayaprakash, Halimah Khalil, Tejal Lathia, Eka Melson, Alisha Narendran, Lynne Robinson, Chitra Selvan, Jameela Sheikh, Saskia Wicks, Nawal Zia, Michael O’Reilly and Wiebke Arlt. All authors contributed to the article and approved the submitted version.

## Supplementary material

Supplementary Figure 1 Network analysis of the relation between various hashtags and “PCOS Hashtags” in September 2021.
