# Supplementary figures and images for: Identifying the challenges and opportunities of PCOS awareness month by analysing its global digital impact
Source: Front Endocrinol (Lausanne). 2023 Mar 2;14:1109141. doi: 10.3389/fendo.2023.1109141 (PMC10018009; doi:10.3389/fendo.2023.1109141)

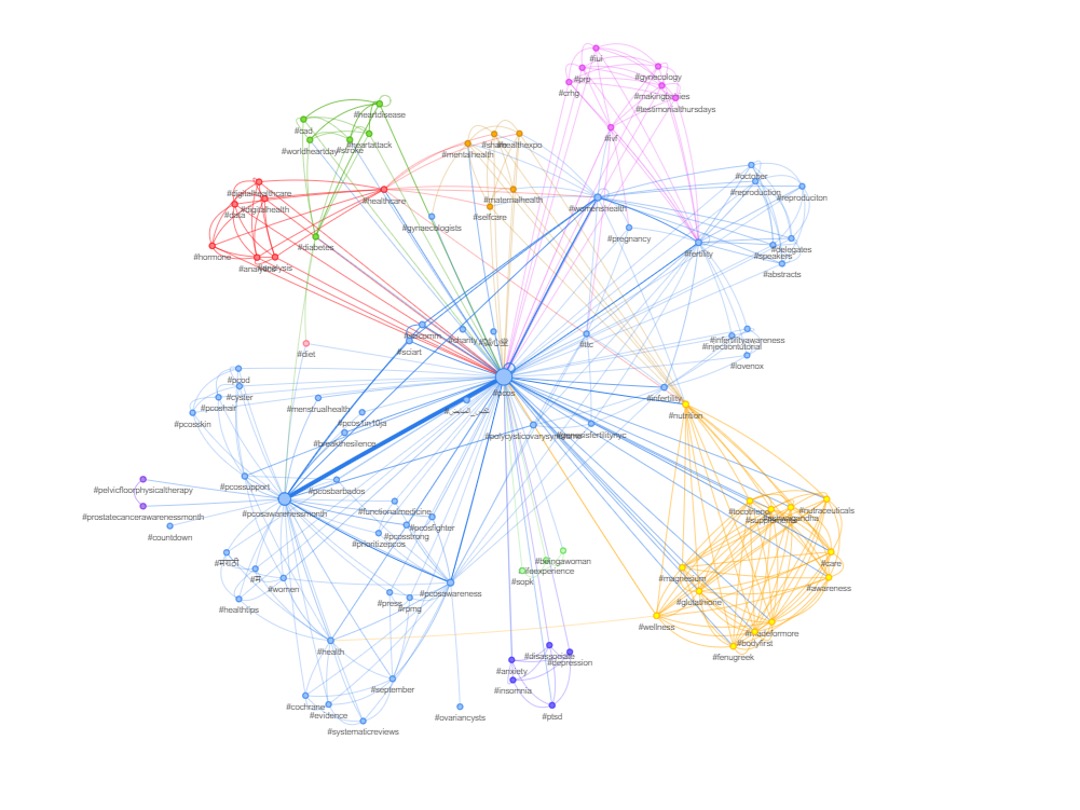

Supplement: Supplementary Figure 1 — Network analysis of the relation between various hashtags and “PCOS Hashtags” in September 2021. [file Image_1.jpeg]
